# Reconstruction-Based Change Detection with Image Completion for a Free-Moving Camera

**DOI:** 10.3390/s18041232

**Published:** 2018-04-17

**Authors:** Tsubasa Minematsu, Atsushi Shimada, Hideaki Uchiyama, Vincent Charvillat, Rin-ichiro Taniguchi

**Affiliations:** 1 Graduate School of Information Science and Electrical Engineering, Kyushu University, 744, Motooka, Nishi-ku, Fukuoka 819-0395, Japan; atsushi@limu.ait.kyushu-u.ac.jp (A.S.); uchiyama@limu.ait.kyushu-u.ac.jp (H.U.); rin@limu.ait.kyushu-u.ac.jp (R.-i.T.); 2 IRIT, Université de Toulouse, CNRS, 31000 Toulouse, France; Vincent.Charvillat@enseeiht.fr

**Keywords:** change detection, background subtraction, convolutional neural network, free-moving camera

## Abstract

Reconstruction-based change detection methods are robust for camera motion. The methods learn reconstruction of input images based on background images. Foreground regions are detected based on the magnitude of the difference between an input image and a reconstructed input image. For learning, only background images are used. Therefore, foreground regions have larger differences than background regions. Traditional reconstruction-based methods have two problems. One is over-reconstruction of foreground regions. The other is that decision of change detection depends on magnitudes of differences only. It is difficult to distinguish magnitudes of differences in foreground regions when the foreground regions are completely reconstructed in patch images. We propose the framework of a reconstruction-based change detection method for a free-moving camera using patch images. To avoid over-reconstruction of foreground regions, our method reconstructs a masked central region in a patch image from a region surrounding the central region. Differences in foreground regions are enhanced because foreground regions in patch images are removed by the masking procedure. Change detection is learned from a patch image and a reconstructed image automatically. The decision procedure directly uses patch images rather than the differences between patch images. Our method achieves better accuracy compared to traditional reconstruction-based methods without masking patch images.

## 1. Introduction

Change detection, which is used in many vision systems, is one of the fundamental techniques for various security tasks, such as video surveillance. Change detection methods can automatically summarize long videos from surveillance cameras to reduce employee workload and the cost for security tasks. The main concept of change detection is to define a background and detect foreground regions as differences between the defined background and a current observation. The background represents a normal or majority state in change detection. For example, a background is typically an image without moving objects. Moving objects are detected as foreground objects when the background image is compared to a current image. Change detection methods can detect objects of any shape, unlike specific object detectors, such as vehicle detectors, because change detection focuses only on image differences without modeling a specific object. This is appropriate for surveillance tasks because foreground regions cannot be defined directly when there are no priors of foreground objects.

Change detection methods are categorized into two methods: those for stationary cameras and those for moving cameras. In stationary camera methods, the background is represented for each pixel based on observed pixel values, which are compared to current pixel values to detect foreground regions via background subtraction [1]. Stauffer et al. [2] estimated a color distribution at each pixel. The estimated distribution was used for the background representation. Stationary camera methods obtain accurate background representations for change detection ignoring camera motion. Recently, drones and robots equipped with cameras have become increasingly available. These moving devices can expand surveillance areas and enhance the flexibility of surveillance systems. Some moving camera methods [3,4] compensate for camera motion to process images from a moving camera. A visual motion estimated between two successive images consists of camera motion, or motion of a moving object. Camera motion is used for background representation because we need not detect camera motion as a foreground in video surveillance. These methods can detect the foreground based on motion differences, but are specialized for moving object detection because of use of camera motion.

The other type of moving camera method, called a one-shot-based method [5,6], is not affected by camera motion. One-shot-based methods prepare background images for target scenes in advance. In a naive implementation of one-shot-based methods, a background image is selected based on appearance similarity between a current image and background images, and the current image is compared to the selected background image for change detection. The one-shot-based methods can also detect stationary objects because of no use of camera motion. This preparation scheme is reasonable for surveillance systems using security robots because robots can routinely observe the backgrounds in a target scene. This naive implementation is strongly affected by instances of background images. For example, many false positives are caused in a detection result when the illumination in a current image is different from one in background images. To solve this problem, we need to estimate a background image from a current image adaptively.

One solution is to reconstruct a background image from a current image. The solution, called a reconstruction-based approach, is a learning-based algorithm [7]. The goal is to obtain a transformation function *f* satisfying f(x)=x, where *x* is an input. The learning procedure uses only background images for the reconstruction. Therefore, foreground regions cannot be accurately reconstructed from current images because foreground regions are not contained in the background images. The foreground regions in a reconstructed image thus create larger errors than background regions. In the detection step, the reconstruction-based approach distinguishes areas with large reconstruction errors as foreground regions.

**Figure 1:**
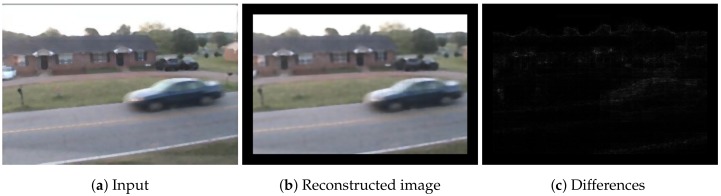
Reconstruction using an autoencoder. We trained the autoencoder using patch images as reference images. We cropped patch images for a current image in (**a**) and reconstructed each patch in (**b**). It is difficult to select a threshold value to distinguish the foreground clearly using the differences, as shown in (**c**).

In the case of a reconstruction algorithm such as an autoencoder [8], errors in foreground regions often have similar values to ones in background regions. Figure 1 presents a reconstructed image by an autoencoder and the differences between the original image and the reconstructed image. We trained the autoencoder in Figure 1 using patch images. Note that another possibility is to reconstruct entire images directly. In fact, we tried the approach, but the reconstructed images were inaccurate. The reconstruction-based methods learn a transformation function f(x)=x using only background images. An ideal *f* is an identity function if the input image contains background regions only. However, such a function causes over-reconstruction when the input image partially contains foreground regions. In other words, foreground regions are also completely reconstructed by the identity function. Therefore, it is necessary to reduce over-reconstruction while keeping the quality of background reconstruction as high as possible. We hypothesize that over-reconstruction of foreground regions is possible through the use of complete patch images, as *f* approximates an identity function in this case. The over-reconstruction causes difficulty in distinguishing the foreground based on the magnitude of differences between a current image and a background image, as illustrated in Figure 1c. Traditional one-shot-based methods use only the magnitude of differences for decision of change detection. In Figure 1, some regions of the house and the edges are background regions. However, errors in the background regions are similar to errors for a car. According to the magnitudes of the errors in Figure 1, the background regions may be detected as foreground regions because of similar errors. Therefore, other criteria are needed for more accurate detection.

We propose a framework of a reconstruction-based change detection method for a free-moving camera. Our method solves over-reconstruction of the foreground region and introduces other criteria for change detection. For our implementation, we combine two convolutional neural networks for image completion and change detection. The image-completion network is applied to a patch image in a current image and generates a reconstructed patch image. The change-detection network then compares the reconstructed patch image to the current patch image to detect changes in appearance. It should be noted that our method is a patch-based method for easily preparing various training images.

**Figure 2:**
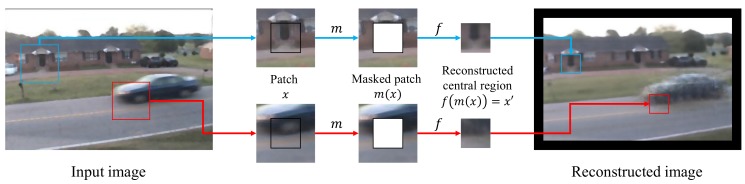
Examples of a reconstruction procedure by our image-completion network. *m* is a function for masking patch images and f is a function for our image-completion. In Figure 2, the moving car is not included in background images used for training our image-completion network. The image-completion network learns how to reconstruct central regions using only background images. Red and blue lines represent reconstruction of a foreground region and a background region, respectively. The background region is reconstructed from a region surrounding the masked central region. A car wheel in the foreground region is not reconstructed in the red line path. The image-completion network cannot use color and texture of the car wheel. Therefore, errors in the car wheel are emphasized.

To solve the over-reconstruction problem, we mask the central regions of patch images and reconstruct these regions from surrounding regions using the image-completion network. Our image-completion network cannot approximate an identity function because the learned function *f* satisfies $f\left(m\left(x\right)\right)=x^{\prime }|m\left(x\right)\neq x^{\prime }$, where m(x) is a masked patch image and $x^{\prime }$ is the central region of *x* as shown Figure 2. The red line in Figure 2 represents reconstruction of a foreground region. A car wheel is masked and the color and the texture of the wheel cannot be used for our reconstruction procedure. The car wheel is not reconstructed because the image-completion network does not learn how to reconstruct the car wheel from the road surface and the front part of the car. As the blue line in Figure 2 illustrates, background regions can be reconstructed more accurately than foreground regions. It is difficult to reconstruct foreground regions from surrounding regions because masked foreground regions negatively affect reconstruction performance.

The change-detection network uses a current patch image and its reconstructed patch image, and estimates a foreground probability of the current patch image. Patch images with foreground labels and background labels are used for supervised learning. As shown in Figure 1, there is a difference between a patch image and the reconstructed patch image, even if the patch image is a background region. The change-detection network learns criteria to accept the differences as background regions through supervised learning. Patch images are used for inputs of the change-detection network. The learning procedure is performed based on the patch images for change detection. Therefore, the change-detection network can obtain criteria in addition to the magnitudes of the differences between the patch images.

## 2. Related Work

It is essential for change detection to define normal situations because “change” is defined by differences from normal situations. Many researchers have discussed what constitutes a normal situation according to various aspects, such as appearance information, including colors and textures, and motion information, including optical flows and the trajectories of feature points. We discuss related studies from the perspectives of stationary and moving camera methods.

### 2.1. Stationary Camera Methods

One efficient approach is background subtraction [1], which was proposed for the computer vision field. Most simple methods of background subtraction define a background image as a normal situation and detect differences in appearance between the defined background image and an observed image. In the case of background subtraction, normal situations are called “background”. Otherwise, they are called “foreground”. These methods attempt to avoid detecting illumination changes based on the time of day, as well as dynamic backgrounds, such as waving trees. However, simple background subtraction detects these changes as foregrounds. Statistical methods [2,9] and case-based methods [10,11] were proposed to model efficient backgrounds for each pixel to handle the problems mentioned above. The definition of a background follows the assumption that background features are frequently observed, while foreground features are rarely observed.

Other background definitions are based on frame-wise information [12] and motion information [13]. Moving objects can be detected from image sequences, as shown in [12]. Background sequences can be represented using a low rank subspace if they change linearly, which is the case in gradual illumination changes. Sequences with foreground regions, such as moving objects, do not satisfy low rank constraints. In [12], a matrix with each row corresponding to a frame in an image sequence was decomposed into a low rank matrix for representing background regions and a sparse matrix for representing foreground regions by using robust principal component analysis. In [13], background dictionaries with prior sparsity were constructed from background features using spatial and temporal information. Therefore, foreground features were not represented by the learned dictionary. In [13], foreground regions were detected based on the errors between an observed feature and a reconstructed feature from a learned dictionary.

Recently, some researchers have proposed approaches using deep neural networks to better utilize feature representations and automatically learn background features. Smeureanu et al. [14] used a pre-trained network for an image classification task to extract higher-level features. They detected anomalies using a one-class support vector machine (SVM) that was trained using features extracted by a pre-trained network. A background subtraction framework using a convolutional neural network (CNN) was proposed in [15]. This CNN-based method could automatically learn background features from training data, unlike previous handcrafted approaches. This method requires background and foreground labels as supervised signals to train a CNN. Autoencoder-based methods were proposed in [7,16] to utilize unsupervised learning methods. The authors of [7,16] used only background regions as training data, which were represented by image sequences, trajectories of feature points, and optical flows. A trained autoencoder cannot clearly reconstruct foreground sequences because it only knows how to reconstruct background sequences, similar to the method in [13]. In [7], change detection was performed by directly comparing input data to reconstructed data. In [16], a one-class SVM was used for learning background features obtained from a trained autoencoder to detect anomalies. However, these studies only discuss the case of using a stationary camera because these methods obtain temporal information from image sequences. Our method also utilizes a framework of deep neural networks to automatically learn background features from training data.

### 2.2. Moving Camera Methods

The main issue in stationary camera methods is that they ignore the selection of new backgrounds after a camera is moved. For example, in [2], a background *i* was modeled based on each pixel *i*, where each pixel *i* always has access to the background *i*. This model collapses in the presence of a moving camera. We must find an appropriate background *x* for the pixel *i* after the camera is moves. Xue et al. [17] proposed an extension of the Gaussian mixture model for a pan-tilt-zoom camera. Gaussian mixture models were constructed using panoramic coordinates projected from image coordinates. Therefore, this method can access appropriate Gaussian mixture models using panoramic coordinates, even if the camera moved. To handle other camera motions, such as translation, other methods have used multiple homographies for different planes [3] and optical flows [4]. These methods could perform registration on continuous frames to find the appropriate background *x*. However, these methods specialize in moving object detection because ego-motion is defined as background content. Our study detects the differences in appearance between a current image and reconstructed background, regardless of the movement of objects.

More general approaches for moving cameras project images captured from various viewpoints into 3D coordinate space and select appropriate background images or features based on 3D positioning. Additional devices utilizing GPS and 3D information, such as 3D models and camera poses, have also been used. In [6], GPS data was used for matching viewpoints in a current image and background image. In [18], a dense 3D model of the target scene was used for the registration of each image in a 3D coordinate space. In [19], the authors used the camera poses of captured images for image localization. These methods are flexible for camera motion, but it is difficult to calibrate certain devices and data. Furthermore, devices are not always available in all circumstances, such as GPS in indoor environments. Our study focuses on using only images with no additional devices and accepting free-moving cameras.

Lawson et al. proposed a case-based approach [5] in which they constructed a background dictionary from only background features. Foreground regions were detected by comparing a current feature to the background features in the dictionary. The computational cost depends on the size of the dictionary because the method must match all background features to the entries in the dictionary. Our method avoids this issue because it reconstructs backgrounds directly from a current image.

## 3. Change Detection with Image Completion

**Figure 3:**
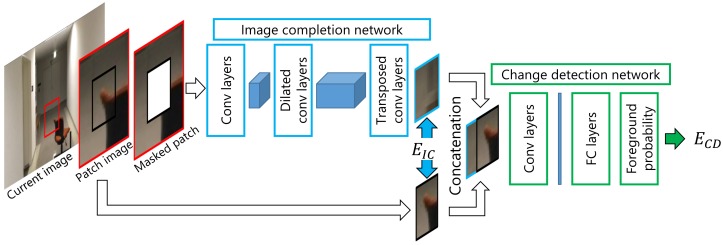
Proposed networks. A patch image is cropped from a current image. After masking the central region of the patch image, the image-completion network receives the masked patch image as an input. The output of the image-completion network and central region of the patch image are concatenated to create the input for the change-detection network. The change-detection network then determines the foreground probability of the patch image.

Our method consists of one network for image completion and another for change detection, as illustrated in Figure 3. A patch image is cropped from a current image and the central region is masked. The masked patch image is passed to the image-completion network to complete the patch image. The image-completion network reconstructs textures and colors in the masked regions. The network learns how to reconstruct backgrounds from training images, which contain only background images. The network does not reconstruct foregrounds such as the chair in Figure 3 because it does not learn any foreground regions. Therefore, foreground regions will have larger differences between the reconstructed image and original patch image compared to background regions. Change detection is performed using the reconstructed patch image and original patch image. Following reconstruction, the reconstructed patch image and original patch image are concatenated as inputs for the change-detection network. The change-detection network classifies the difference between the original patch image and reconstructed patch image as a background or foreground.

### 3.1. Network for Image Completion

Our method is inspired by image completion methods from Pathak et al. [20] and Satoshi et al. [21]. These authors performed an estimation of realistic textures and colors in a masked region selected by a user. Their methods estimated the realistic images based on the context and semantics of real images. To reconstruct backgrounds, our network uses the structure for a completion network proposed by Satoshi et al. [21]. A completion network based on a fully convolutional network consists of dilated convolution layers [22] and transposed convolution layers. The dilated convolution operation resizes the kernel of a traditional convolution operation by padding k-1 zeros between each element of the kernel, where *k* is the dilation rate. A dilated convolution layer can perform a convolution operation for large regions of inputs without losing input resolution. Dilated convolution layers work more efficiently for learning the features of regions without masks compared to traditional convolution layers.

Our completion network learns how to reconstruct background images during the training phase. Pathak et al. and Satoshi et al. used generative adversarial networks (GANs) [23] to obtain realistic images. However, the purpose of the completion networks by Pathak et al. and Satoshi et al. was not to obtain true background images from the training images. Therefore, the terms for the GAN are removed from the cost function and only the reconstruction errors of background images are used in our cost function $E_{\mathrm{IC}}$. In our study, a central region of input *I* is filled with a specific value and our network reconstructs this central region, called $I_{c}$, as follows:

(1) $$E_{\mathrm{IC}}\left(f\right)=\sum \left|f\left(I_{m}\right)-I_{c}\right|,$$

where $I_{m}$ is a patch image *I* whose central region is masked by a specific value, $f(I_{m})$ is the estimated central region, and |·| represents the L1 distance. The size of $I_{m}$ is w×w and the size of $I_{c}$ is 0.5w×0.5w. The cost function is similar to that for an autoencoder. However, the textures and colors in the central region are not used for the reconstruction of the central region, unlike the autoencoder.

### 3.2. Network for Change Detection

A naive change detection method is to set a threshold for the magnitude of differences between $f(I_{m})$ and $I_{c}$ directly. However, this naive method may detect differences as changes in background regions incorrectly when the magnitudes of differences in the backgrounds regions are similar to those in the foreground regions.

We use a background subtraction framework based on a convolutional neural network [15]. The network detects changes based on not only the differences between a current image and its corresponding background image but also the context of the two images. For example, shadows are defined as backgrounds in video surveillance systems. Shadow regions cause differences between a current image and its corresponding background image, as with foreground regions. False positives occur in shadow regions when change detection is performed based on only evaluating the differences. The network was able to classify shadow regions into backgrounds because the network learned shadows as backgrounds through supervised learning [15]. Therefore, the network can perform change detection based on more efficient criteria for target scenes than change detection methods based on only a simple strategy such as thresholding of the difference.

This method requires pre-defined background images, which are created using the temporal median filter proposed in [15], for each scene. Images reconstructed by the image-completion network are used instead of pre-defined background images. Our network works even if a camera moves because our method facilitates both image completion and change detection.

In each convolution layer and fully connected layer, other than the final layer, we use the rectified linear unit (ReLU) activation function. A sigmoid function is used in the final layer to obtain a foreground probability. We also use a cross-entropy function, similar to [15], for training the weights W of the network.

(2) $$E_{\mathrm{CD}}\left(g\right)=-\sum \left(t\log\left(g\left(x\right)\right)+\left(1-t\right)\log\left(1-g\left(x\right)\right)\right)+0.1{\left|W\right|}^{2},$$

where x is the input created by concatenating $f(I_{m})$ with $I_{c}$ along an axis of channels, *t* is a supervised signal for the input, and g(x) is the foreground probability in x. ${\left|W\right|}^{2}$ is an L2 regularization term for avoiding overfitting.

### 3.3. Training Data

According to Equation (2), to train the change-detection network, training images must contain regions of both background and foreground classes because the network is based on a supervised learning strategy. However, we cannot prepare foreground class images because we cannot define any foreground class. A previous article [24] reported that a change-detection network could detect cars when it was trained using training images containing people as foreground objects. The training images did not contain cars as foreground objects. This implies that a change-detection network can learn what changes are without depending on the shapes and colors of foregrounds in the training images.

Based on this insight, foregrounds from other scenes were used to train our change-detection network. We prepared two types of training images: target images to learn the reconstruction of backgrounds in a target scene, and detection images to learn differences. The target images do not contain any foregrounds, while the detection images contain both foregrounds and backgrounds. To minimize Equation (1), only background images from the target images and detection images are used by the image-completion network. Additionally, we minimize Equation (2) by using all training images from both the target images and detection images to train the change-detection network.

### 3.4. Training

Initially, the image-completion network is trained with a mini-batch size of 24 training samples over 10 epochs. The mini-batches consist of 16 patch images from the target images and eight patch images from the backgrounds of the detection images. The change-detection network is then trained using the reconstructed images with a mini-batch size of 32 training samples over 10 epochs. The mini-batches consist of 16 patch images from the target images and eight patch images from the backgrounds and foregrounds of the detection images. Patch images with rare textures among the training images make it more difficult to train our networks. After training the change-detection network, the foreground probability is computed for each patch image among the target images. Patch images with a foreground probability greater than 0.1 are stored as challenging images. We train our networks again over 10 epochs by randomly selecting 32 data samples from the training images and challenging images. The challenging images thus have a better chance of being learned by our networks.

The filters in our network are initialized using a Xavier initialization [25]. We use an ADAM optimizer [26] to train our networks with parameters $\beta_{1}=0.9$, $\beta_{2}=0.999$, and a learning rate of 0.0001. RGB color images are used as inputs. Pixel intensities are divided by 255 to obtain normalized intensities between 0 and 1. We used 2.0 as the specific value for masking a central region in the patch image because the value 2.0 is not included in the range of the normalized intensities.

## 4. Experimental Setting

### 4.1. Dataset

One type of surveillance system discovers changes based on differences between an observation from a particular day and observations from the past several days. In such a surveillance system, detected changes representing situations requesting caution are communicated to security officers. The surveillance system requires observations from the past several days to contain only background images to define a background for a target scene. There are many datasets available for change detection based on images. However, few open-source datasets contain pure background images of training data for moving cameras.

**Figure 4:**
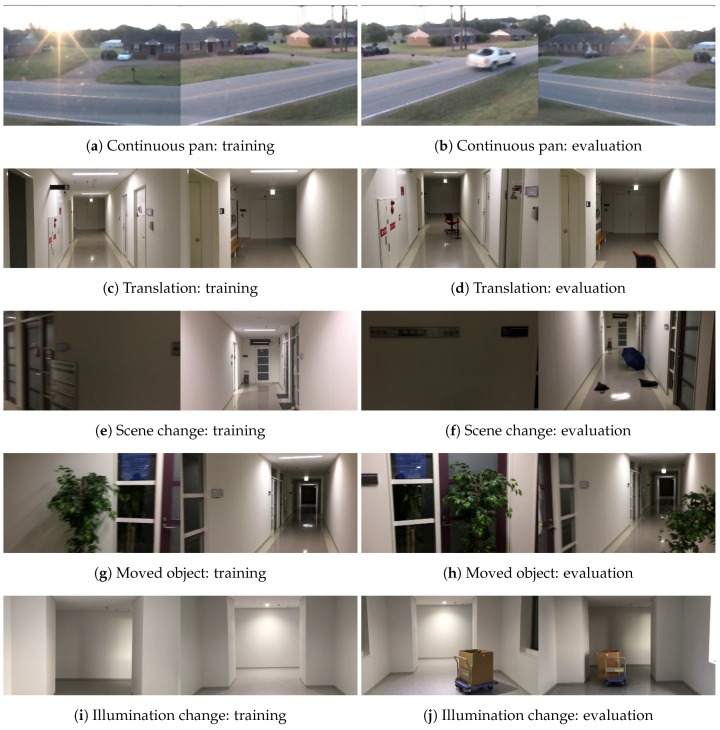
Example images from our dataset.

We created a new dataset for our experiments based on the characteristics of the surveillance system. The training data in our dataset includes only background images to learn the characteristics of backgrounds, and the evaluation data has viewpoints similar to the training data. Additionally, changes between the training data and evaluation data are caused by new objects or moved objects in our dataset. Our dataset contains four image sequences we captured and one sequence provided by Changedetection.net [27]. Figure 4 presents examples from our dataset and Table 1 contains a summary of the configurations in the dataset. We created patch images with a stride of 16 and patch size of 64×64. We shifted the location of cropping by ±3 and ±7 pixels along the *x*- and *y*-axes, respectively, as a method of data augmentation when we cropped the patch images.

Detailed descriptions of the sequences are provided below:

- **Continuous pan:** This image sequence came from [27] and was captured by a panning camera. Moving cars appear as foreground objects in this sequence. We divided this sequence into two sub-sequences for training and evaluation. To obtain training data without moving cars, we dropped frames with moving cars from the sub-sequence for training.
- **Translation:** A camera moves straight ahead in this sequence, unlike in the continuous pan sequence. This sequence was captured in a corridor. The evaluation data contains an orange chair as a foreground object and the chair does not appear in the training data.
- **Scene change:** We turned a corner in a corridor to capture this sequence. After turning the corner, the appearance of the scene is very different. Using the appearance information from before turning the corner is not effective after turning the corner. In the evaluation data, foreground objects are umbrellas and the training data do not contain the umbrellas.
- **Moved object:** This sequence contains potted trees in both the training data and evaluation data. However, the positions of the potted trees are different between the training data and evaluation data. It may be difficult to detect moved potted trees as foreground objects if a method learns potted trees as background objects. In this sequence, the moved potted trees are considered to be foreground objects, as described above.
- **Illumination change:** After capturing training data for the above sequences, we captured evaluation data immediately afterwards. In contrast, the training data and evaluation data for this sequence were captured on different days. Environments change based on the sunlight during the day and fluorescent lights at night. In this sequence, boxes on a cart are foreground objects.

We used foreground images provided by Changedetection.net [27] for the detection images described in Section 3.4 to train the change-detection network. Changedetection.net also provides pixel-wise manually labeled ground truth data. The supervised signals that are used to train the change-detection network are provided for each patch image because the network provides a foreground probability for each patch. Background labels are provided for patch images that do not contain foregrounds. Foreground labels are given to patch images that contain foreground objects in the centers of the patch images. We chose approximately 100 images from “highway” and “pedestrians” sets for training. We created patch images with a stride of 4 and patch size of 64×64. We resized the images to H×W, 0.8H×0.8W, 0.6H×0.6W, and 0.4H×0.4W for data augmentation, where *H* and *W* are the height and width of the original images, respectively. The chosen images contain cars and pedestrians as foreground objects. Our dataset for the continuous pan sequence also contains moving cars, but the sizes of the cars vary significantly.

### 4.2. Compared Methods

We compare existing methods using only information from images to the proposed method. However, there are a few existing methods to detect foregrounds from a single image. To the best of our knowledge, nearly all existing methods use stationary cameras and temporal information obtained from multiple frames. Therefore, for the sake of comparison, we modify existing methods to detect foregrounds from a single image.

The baseline method for comparison is a case-based method. It uses a dictionary constructed from background patch images as training data. To detect foregrounds, we provide a test patch image and search for candidates in the corresponding background patch images from the constructed dictionary. We compute the sum of squared differences (SSD) between the test patch image and candidates, and then select the background patch image with the lowest SSD among the candidates. The foregrounds are then detected by setting a threshold on pixel-wise differences between the test patch image and nearest candidate. We constructed the dictionary by using a local sensitive hash to search through candidates for background patch images efficiently. We used a 10-bit hash code with cosine distance. Additionally, we used principal component analysis (PCA) to obtain image representations for the local sensitive hash. The dimension of representation is determined by the number of principal components to retain 99% of the total variance. We chose 100,000 patch images for PCA based on the limitations of our PC memory. This method is denoted as CASEBASED.

A one-class SVM-based anomaly detection method was proposed in [14] for frame-level and pixel-level detection. We modified the detection strategies because the original method leveraged multiple frames for the localization of detected changes. We applied patch images to the method to obtain detection results for each patch region. For each patch image, we calculate the signed distance from the hyperplane learned by the SVM and threshold the distance for the detection of foregrounds. In [14], a pre-trained VGG-f [28] was used for feature extraction. The VGG-f was trained using the ILSVRC benchmark [29] and extracted superior image representations for classification tasks. We utilized the VGG-f provided by the Caffe Model Zoo. Our parameter settings follow those defined in [14]. To use large-scale data to train our one-class SVM classifier, we used a random sampling consensus (RANSAC)-based method [30] for optimization. Note that the computational complexity for training SVMs depends on the cube of the number of training data samples *N* for a naive implementation [31]. We apply the methodology for RANSAC-SVM proposed in [31] to train our one-class SVM classifier. We briefly describe the training procedure below. We first optimize the classifier using a randomly selected subset of training data. We then evaluate the classifier on all of the training data. We conclude the training procedure if the number of training data classified as anomalies is approximately equal to νN, where ν is the regularization parameter for the one-class SVM and νN training data are accepted as outliers. Otherwise, we update the subset and optimize the classifier using the updated subset iteratively until the condition is satisfied or the number of iterations reaches a user-defined value. We reduce the computational time for training because the size of the subset is much smaller than the size of the entire training dataset. We set the number of samples in a subset to 1000. This method is denoted as OCSVM.

The autoencoder-based anomaly detection method (AE) was first proposed in [7]. In [7], multiple frames were used as inputs to the autoencoder to leverage spatial and temporal information. In our preliminary experiments, we attempted to utilize only a single frame as the input to the autoencoder. However, the trained autoencoder provided low-quality reconstructed images in the case of a moving camera. One of the reasons for this is the diversity of viewpoints of a moving camera. The trained autoencoder reconstructed background regions accurately and eliminated foreground objects when the viewpoints of the camera were limited, such as the viewpoints when using a stationary camera or pan-tilt camera. In this experiment, we utilized patch images cropped from one observed image as the input for the autoencoder to reduce differences in appearance caused by the diversity of viewpoints. We considered two methods to calculate differences between a current patch image and a reconstructed patch image. The first method simply computes the Euclidean distance (ED) between the two patch images based on the RGB value of each pixel. The second method uses the change-detection network (CDNET), as described in Section 3.2. After executing each method, we applied a threshold to the output to obtain a binary image as a final result. The two methods are denoted as AE-ED and AE-CDNET, respectively. The architecture of the autoencoder is described in Table 2.

The architectures of the image-completion network (ICNET) and the change-detection network are also described in Table 3. We compared two versions of our method (ICNET-ED and ICNET-CDNET) to evaluate the effectiveness of the change-detection network. ICNET-ED uses ED instead of the change-detection network to compare a current patch image and reconstructed patch image from the image-completion network, similar to AE-ED. ICNET-CDNET includes all of the procedures described in Section 3.

In all compared methods, as well as the proposed method, the stride of the sliding window for cropping patch images from a frame was 16. The results of the overlapped regions were averaged before applying a threshold to obtain a final decision for each method.

### 4.3. Evaluation Metric

We used precision, recall, and F-measure as performance metrics to evaluate the accuracy of foreground detection results. These metrics are defined as follows:

(3) $$\mathrm{Precision}=\frac{\mathrm{TP}}{\mathrm{TP}+\mathrm{FP}},\mathrm{Recall}=\frac{\mathrm{TP}}{\mathrm{TP}+\mathrm{FN}},F-\mathrm{measure}=\frac{2\times \mathrm{Precision}\times \mathrm{Recall}}{\mathrm{Precision}+\mathrm{Recall}},$$

where true-positive (TP) is the number of pixels correctly detected as foreground, false-positive (FP) is the number of pixels incorrectly detected as foreground, and false-negative (FN) is the number of pixels incorrectly detected as background. We manually constructed bounding boxes for the ground truth in all frames and used them in our evaluations. Pixels inside of bounding boxes are foreground pixels.

We modified the threshold value to evaluate the precision–recall curves. CASEBASE, AE-ED, and ICNET-ED are based on a comparison of Euclidean distances in the RGB color space. These methods used the same set of threshold values {10,20,...,90}. The outputs of AE-CDNET and ICNET-CDNET are probabilities of the foreground class. We used {0.1,0.2,...,0.9} as the set of threshold values in AE-CDNET and ICNET-CDNET. OCSVM uses the signed distances during the training phase as threshold values. We computed the signed distances from the subset of training data and selected the *p*-th percentile of the computed signed distance as the threshold value. We set p={2,4,...,20}.

## 5. Results

**Figure 5:**
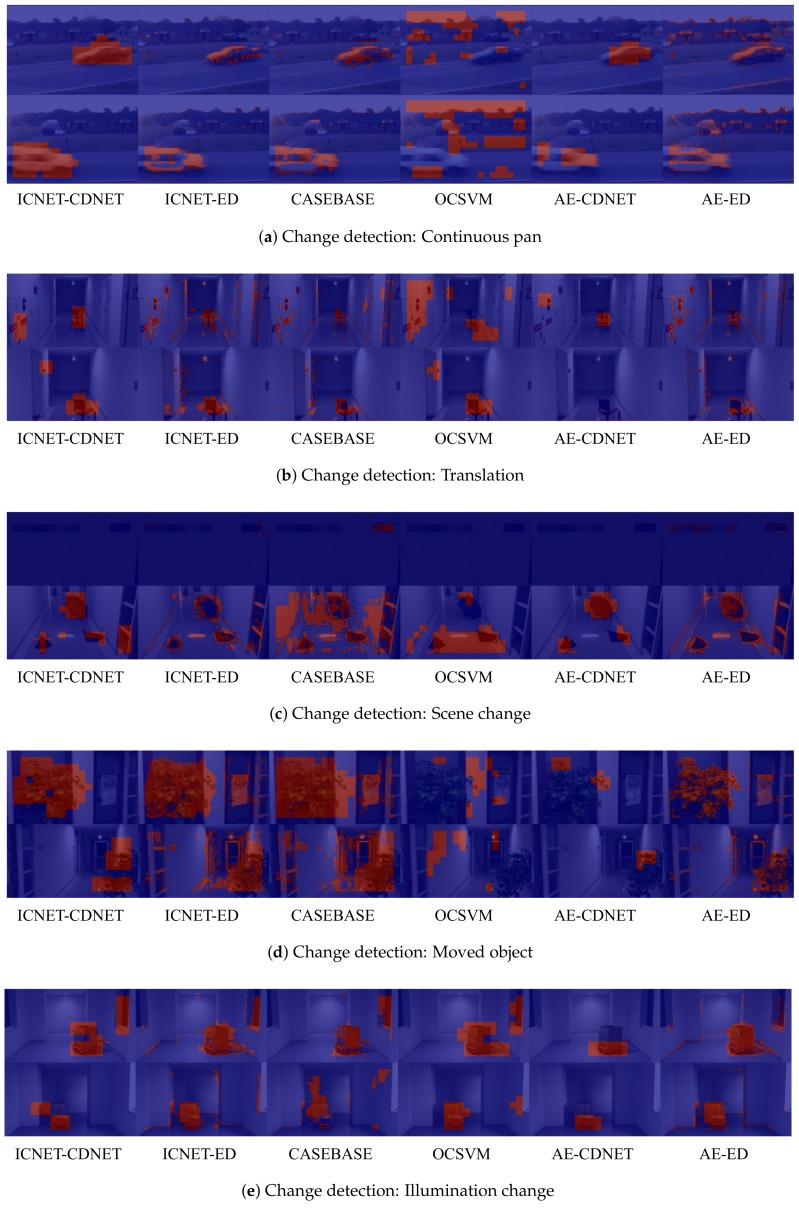
Results of change detection using different methods. Resulting images represent the input images overlaid with red and blue pixels to represent foreground and background pixels, respectively.

**Figure 6:**
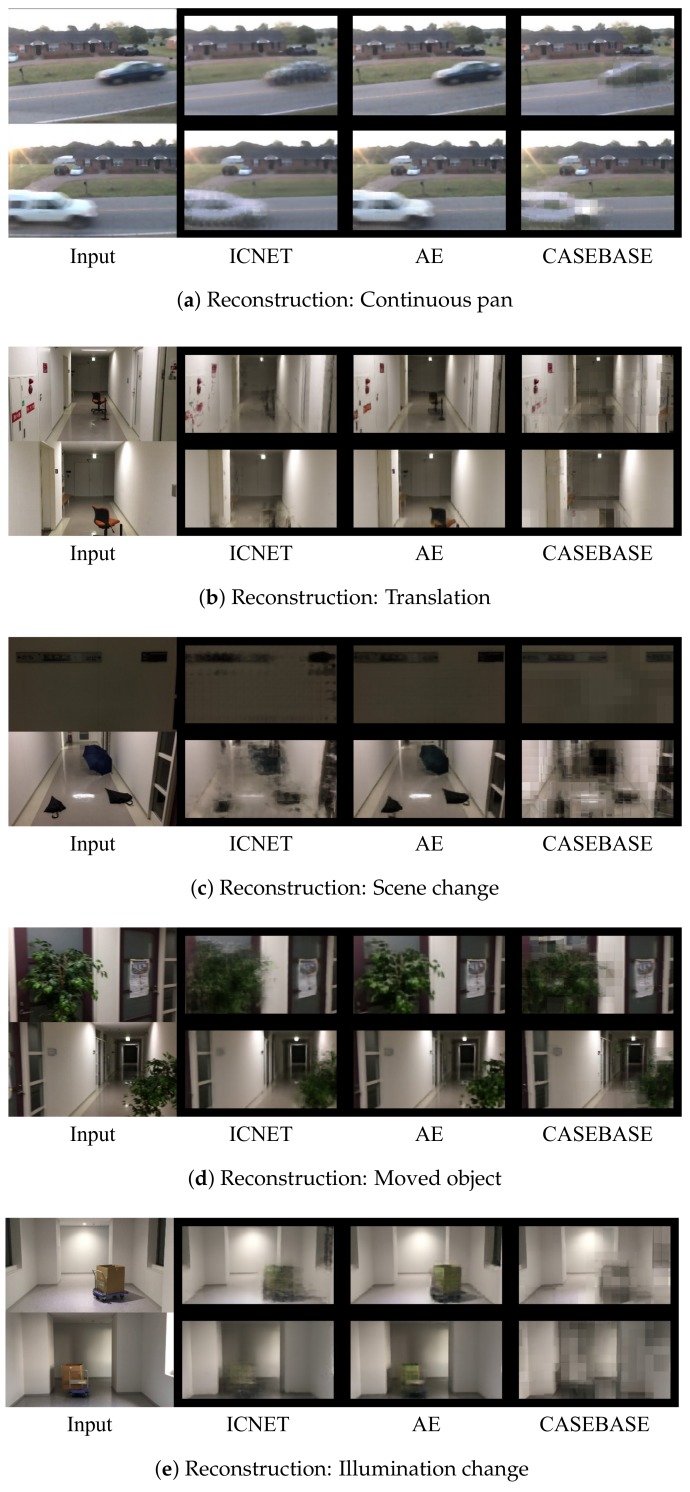
Reconstructed images using different methods. The reconstructed images in CASEBASE are derived from patch images obtained from a dictionary. The reconstructed images were used to obtain the results shown in Figure 5 with the input image (first column), ICNET (second column), AE (third column), and CASEBASE (fourth column).

We evaluated both of our methods and the existing methods using the five sequences described in Section 4.1. Some examples of the results of each method are presented in Figure 5. Additionally, Figure 6 illustrates the reconstructed images from ICNET, AE, and CASEBASE. Note that CASEBASE is not a reconstruction-based method, but obtains patch images of backgrounds from the dictionary for change detection. Figure 6 presents the obtained images for CASEBASE.

**Figure 7:**
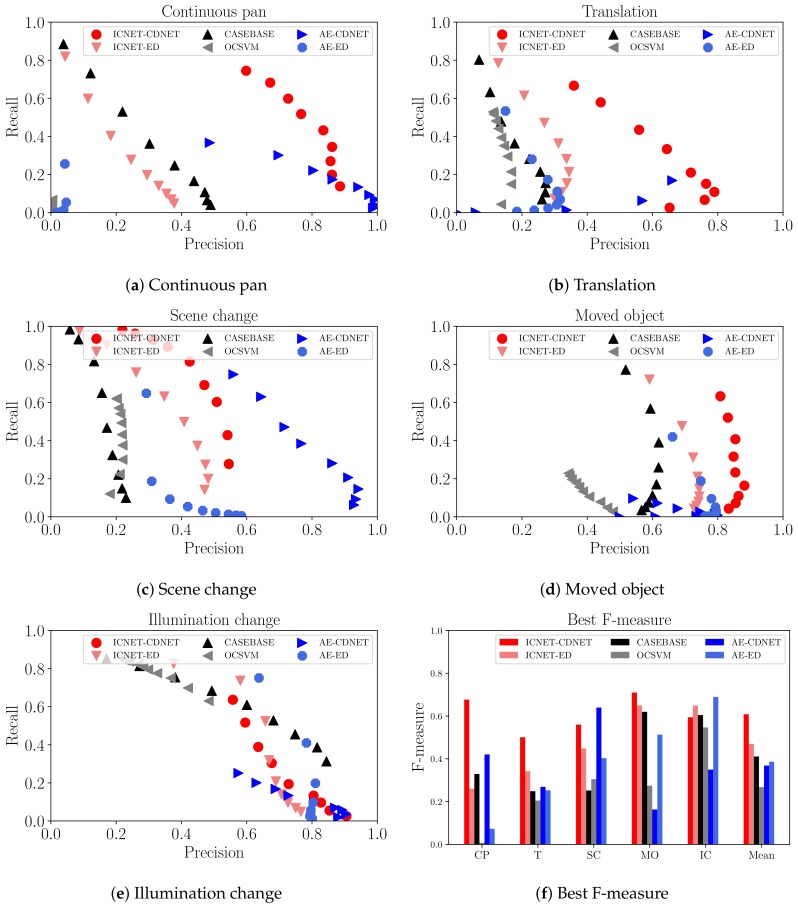
Evaluation on precision–recall curve and Best F-measures. We use abbreviated styles for the names of our datasets: continuous pan (CP), translation (T), scene change (SC), moved object (MO), and illumination change (IC).

Firstly, we discuss the accuracy of the foreground detection results of each method in terms of precision, recall, and F-measure. We then investigate the changes in accuracy caused by the effects of image completion. For a quantitative evaluation of the results in these experiments, we list the best F-measure for each sequence in Table 4. Additionally, precision–recall curves for the varying threshold values are presented in Figure 7.

### 5.1. Quantitative Comparison

Table 4 shows that our methods (ICNET-CDNET and ICNET-ED) yielded more accurate results than AE-CDNET and AE-ED. ICNET-CDNET and ICNET-ED demonstrated the best and second-best performance for the “Mean” in Table 4. Comparing ICNET-CDNET with ICNET-ED, CDNET can improve accuracy further than ED because of its ability to learn efficient criteria for target scenes in change detection using a current image and reconstructed image. We discuss the image-completion effect when comparing ICNET to AE in Section 5.2. Our method detected changes in spite of moving objects and stationary objects. Additionally, the dataset used in the experiments contains some types of camera motion such as rotation and translation. Therefore, our method can work in a free-moving camera.

CASEBASE provided the third-best performance for the “Mean” in Table 4. According to Table 4, the recall of CASEBASE is higher than the precision. This indicates that CASEBASE did not identify patches with similar appearances from the training data in some background regions. We observed that appropriate patches were not identified in the sequence of illumination change images. Figure 5e and Figure 6e present failure cases for CASEBASE. The sequence of illumination change images contains different lighting conditions between the training data and evaluation data. Therefore, it is difficult to obtain appropriate patches from the training data. Reconstruction-based methods such as AE and ICNET can ameliorate this problem, as shown in Figure 6e. AE and ICNET could successfully reconstruct backgrounds, even if identical patch images were not contained in the training data, as shown in Figure 6e.

In our experiments, OCSVM provided the lowest accuracy among all compared methods. OCSVM considers a portion of the backgrounds in the training data as outliers. However, there are no outliers in our training data. OCSVM always detected certain regions in the training data as foregrounds incorrectly. Therefore, it had difficulty learning reasonable discriminative planes in our experiments, except for illumination change images. In the sequence of illumination change images, OCSVM could learn a discriminative plane easily because this sequence has a low diversity of colors, including mainly white walls and brown boxes.

ICNET experienced misdetections when a central region in the patch image was not uniquely reconstructed from the surrounding regions. For example, ICNET could not reconstruct a small red notice on a white wall in the translation sequence, as shown in Figure 6b. ICNET could not determine if there was only a white wall or a small red notice on a white wall from the masked patch image. ICNET-CDNET and ICNET-ED incorrectly detected the small red notice as a foreground object because the reconstructed image containing the small red notice had a large number of errors.

### 5.2. Effects of Image Completion

**Figure 8:**
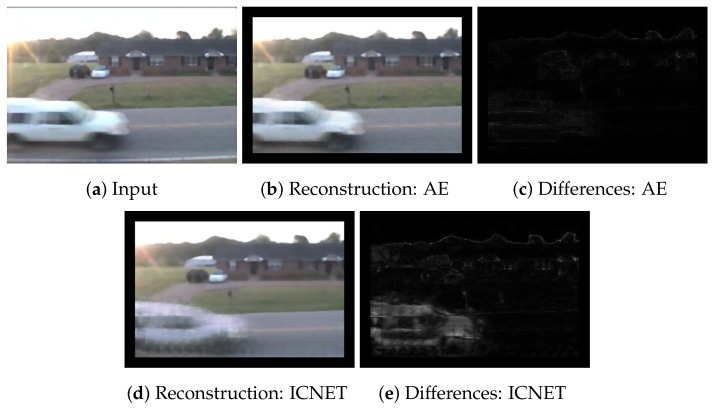
Comparison of reconstruction performances. The reconstructed image from ICNET (**d**) has a larger number of differences in the foreground region than AE (**b**). These larger differences lead to a larger recall in ICNET-ED, as shown in Figure 7.

According to Figure 7, the recall of ICNET-ED was higher than that of AE-ED when ICNET-ED and AE-ED used the same threshold parameter. This means that reconstructed images from ICNET have larger differences between current images and reconstructed images than AE. We present examples of reconstructed images from ICNET and AE in Figure 6 and Figure 8, respectively. AE can reconstruct foreground regions more clearly than ICNET, as shown in Figure 6. The differences in ICNET were enhanced in the foregrounds, as shown in Figure 8. Therefore, ICNET improved the accuracy of change detection.

We used CDNET after reconstructing an image using ICNET and AE. This was done to use not only the magnitudes of the differences caused by reconstruction, but also patterns for change detection. ICNET-CDNET improved the accuracy compared with ICNET-ED. As mentioned above, ICNET improved recall, but did not contribute to improving precision. Therefore, ICNET-CDNET worked to suppress misdetections. However, AE-CDNET did not always contribute to improving accuracy. In the sequence of continuous pan images, AE-CDNET worked well for detection improvement. CDNET for AE successfully learned the differences caused by reconstruction of backgrounds during training. For the sequence of moved object images, the accuracy of AE-CDNET was worse than AE-ED. The sequence of moved object images contains the same objects (potted trees) as backgrounds and foregrounds. Note that the textures behind the potted trees change from a white wall to a door or corridor because the positions of the potted trees are different between the training data and evaluation data. CDNET for AE could not distinguish the differences in moved potted trees between a current image and reconstructed image because the differences from the AE were small and the CDNET learned the small differences in potted trees as background regions during the training step. The combination of AE-CDNET is not efficient for change detection when a target sequence has similar objects in the training data and evaluation data. ICNET enhanced the differences between moved potted trees because the textures in regions of the moved potted trees change. The differences in moved potted trees did not appear in the training step. Therefore, ICNET-CDNET is the superior combination for change detection.

## 6. Conclusions

In order to handle any type of camera motion in surveillance systems, we proposed a reconstruction-based change detection method comprised of one network for image completion and another for change detection. The image-completion network reconstructs a central region from surrounding regions in a patch image. The central region of the patch image is masked before it is applied to the image-completion network to enhance the differences in subsequent reconstructed foreground regions. The change-detection network compares the reconstructed image to a current image to detect differences in foreground regions only. Criteria for target scenes are learned from patch images and the reconstructed patch images automatically. Our method achieved the best performance among all compared methods and yielded a better F-measure in terms of detection accuracy than reconstruction methods based on autoencoders without the masking of patch images.

Reconstruction by the image-completion network contains ambiguity. This ambiguity leads to misdetection because of incorrect reconstructions of background regions. To solve this issue, we will use the information from a complete image in addition to the masked patch images for the reconstruction procedure in future research. We expect that the accuracy of reconstruction and change detection will be improved by using a network with information from a complete image.

The change-detection network improved detection accuracy in our experiments. Details of criteria learned by the network were not discussed in this paper. The analysis helps to increase the network performance and understand the network behavior. We will analyze the efficient criteria as future work.

References

## Figures and Tables

**Table 1:** Summary of our dataset.

Name	Foreground	Training Frames	Evaluation Frames	Resolution
Continuous pan	Moving cars	232	500	352×240
Translation	Orange chair	350	309	320×180
Scene change	Umbrellas	501	267	320×180
Moved object	Moved potted trees	235	101	320×180
Illumination change	Box and cart	240	58	320×180

**Table 2:** Architecture of the autoencoder. “Stride” is the rate of up/down sampling. When “Stride” is 2×2, the size of the activation map is reduced by half. “fc” in Type indicates a fully connected layer.

Type	Kernel	Stride	${\mathrm{Channel}}_{\mathrm{out}}$
conv	3×3	2×2	64
conv	3×3	2×2	128
conv	3×3	2×2	256
conv	3×3	2×2	512
conv	2×2	1×1	512
fc	-	-	512
transposed conv	1×1	1/2×1/2	512
transposed conv	3×3	1/2×1/2	256
transposed conv	3×3	1/2×1/2	128
transposed conv	3×3	1/2×1/2	64
transposed conv	3×3	1/2×1/2	3

**Table 3:** Architecture of our networks. “Stride” is the rate of up/down sampling. When “Stride” is 2×2, the size of the activation map is reduced by half. “fc” in Type indicates a fully connected layer. In the “conv” type of (b), the “Kernel” is 2×2 and the “Stride” is 1×1. In the “maxpooling” type of (b), the “Kernel” is 2×2 and the “Stride” is 2×2.

(a) Architecture of ICNET					(b) Architecture of CDNET	
**Type**	**Kernel**	**Dilation Rate** k	**Stride**	${\mathrm{Channel}}_{\mathrm{out}}$	**Type**	${\mathrm{Channel}}_{\mathrm{out}}$
conv	3×3	1	2×2	32	conv+maxpooling	64
conv	3×3	1	2×2	64	conv+maxpooling	128
conv	3×3	1	1×1	128	conv+maxpooling	256
dilated conv	3×3	2	1×1	128	fc	512
dilated conv	3×3	4	1×1	128	fc	256
conv	3×3	1	1×1	256	fc	128
conv	3×3	1	1×1	256	fc	1
transposed conv	1×1	1	1/2×1/2	128		
conv	3×3	1	1×1	64		
conv	3×3	1	1×1	32		
conv	3×3	1	1×1	3		

**Table 4:** Detection accuracy in terms of precision (P), recall (R), and F-measure (F) based on the best parameters for the F-measure. The red and blue numbers denote the best and second-best score in each sequence, respectively.

Method	Continuous Pan			Translation			Scene Change		
	P	R	F	P	R	F	P	R	F
ICNET-CDNET	0.672	0.682	0.677	0.441	0.579	0.501	0.470	0.691	0.560
ICNET-ED	0.246	0.278	0.261	0.269	0.472	0.342	0.408	0.498	0.449
CASEBASE	0.302	0.362	0.329	0.223	0.282	0.249	0.156	0.651	0.252
OCSVM	0.004	0.067	0.008	0.145	0.351	0.205	0.213	0.540	0.305
AE-CDNET	0.698	0.301	0.421	0.661	0.169	0.269	0.559	0.748	0.640
AE-ED	0.043	0.256	0.073	0.231	0.280	0.253	0.292	0.648	0.403
**Method**	**Moved Object**			**Illumination Change**			**Mean**		
	**P**	**R**	**F**	**P**	**R**	**F**	**P**	**R**	**F**
ICNET-CDNET	0.808	0.633	0.710	0.557	0.636	0.594	0.590	0.644	0.608
ICNET-ED	0.591	0.721	0.649	0.580	0.736	0.649	0.419	0.541	0.470
CASEBASE	0.518	0.772	0.620	0.600	0.609	0.605	0.360	0.535	0.411
OCSVM	0.343	0.229	0.275	0.483	0.630	0.547	0.237	0.363	0.268
AE-CDNET	0.541	0.096	0.163	0.575	0.251	0.350	0.607	0.313	0.369
AE-ED	0.661	0.419	0.513	0.638	0.751	0.690	0.373	0.471	0.386

## References

[1] Bouwmans T. (2014). Traditional and recent approaches in background modeling for foreground detection: An overview. Comput. Sci. Rev..

[2] Stauffer C., Grimson W.E.L. Adaptive background mixture models for real-time tracking. Proceedings of the IEEE Computer Society Conference on Computer Vision and Pattern Recognition.

[3] Zamalieva D., Yilmaz A., Davis J.W. (2014). A Multi-transformational Model for Background Subtraction with Moving Cameras. Computer Vision—ECCV 2014.

[4] Lim J., Han B. (2014). Generalized Background Subtraction Using Superpixels with Label Integrated Motion Estimation. Computer Vision—ECCV 2014.

[5] Lawson W., Hiatt L., Sullivan K. Detecting anomalous objects on mobile platforms. Proceedings of the IEEE Conference on Computer Vision and Pattern Recognition Workshops.

[6] Sakurada K., Okatani T. Change Detection from a Street Image Pair using CNN Features and Superpixel Segmentation. Proceedings of the BMVC.

[7] Hasan M., Choi J., Neumann J., Roy-Chowdhury A.K., Davis L.S. Learning temporal regularity in video sequences. Proceedings of the IEEE Conference on Computer Vision and Pattern Recognition.

[8] Hinton G.E., Salakhutdinov R.R. (2006). Reducing the dimensionality of data with neural networks. Science.

[9] Elgammal A., Harwood D., Davis L. (2000). Non-parametric model for background subtraction. Computer Vision-ECCV 2000.

[10] Barnich O., Van Droogenbroeck M. ViBe: A powerful random technique to estimate the background in video sequences. Proceedings of the IEEE International Conference on Acoustics, Speech and Signal Processing, ICASSP 2009.

[11] Kim K., Chalidabhongse T.H., Harwood D., Davis L. (2005). Real-time foreground—Background segmentation using codebook model. Real-Time Imaging.

[12] Candès E.J., Li X., Ma Y., Wright J. (2011). Robust principal component analysis?. J. ACM.

[13] Lu C., Shi J., Jia J. Abnormal event detection at 150 fps in matlab. Proceedings of the IEEE International Conference on Computer Vision.

[14] Smeureanu S., Ionescu R.T., Popescu M., Alexe B., Battiato S., Gallo G., Schettini R., Stanco F. (2017). Deep Appearance Features for Abnormal Behavior Detection in Video. Image Analysis, Part II, Processing of the 19th International Conference, ICIAP 2017, Catania, Italy, 11–15 September 2017.

[15] Braham M., Van Droogenbroeck M. Deep background subtraction with scene-specific convolutional neural networks. Proceedings of the 2016 IEEE International Conference on Systems, Signals and Image Processing (IWSSIP).

[16] Xu D., Yan Y., Ricci E., Sebe N. (2017). Detecting anomalous events in videos by learning deep representations of appearance and motion. Comput. Vis. Image Underst..

[17] Xue K., Liu Y., Ogunmakin G., Chen J., Zhang J. (2013). Panoramic Gaussian Mixture Model and large-scale range background substraction method for PTZ camera-based surveillance systems. Mach. Vis. Appl..

[18] Taneja A., Ballan L., Pollefeys M. Image based detection of geometric changes in urban environments. Proceedings of the 2011 IEEE International Conference on Computer Vision (ICCV).

[19] Sakurada K., Okatani T., Deguchi K. Detecting Changes in 3D Structure of a Scene from Multi-view Images Captured by a Vehicle-Mounted Camera. Proceedings of the IEEE Conference on Computer Vision and Pattern Recognition (CVPR).

[20] Pathak D., Krähenbühl P., Donahue J., Darrell T., Efros A. Context Encoders: Feature Learning by Inpainting. Proceedings of the Conference on Computer Vision and Pattern Recognition (CVPR).

[21] Iizuka S., Simo-Serra E., Ishikawa H. (2017). Globally and Locally Consistent Image Completion. ACM Trans. Gr..

[22] Yu F., Koltun V. Multi-Scale Context Aggregation by Dilated Convolutions. Proceedings of the ICLR.

[23] Goodfellow I., Pouget-Abadie J., Mirza M., Xu B., Warde-Farley D., Ozair S., Courville A., Bengio Y. Generative adversarial nets. Proceedings of the 27th International Conference on Neural Information Processing Systems.

[24] Minematsu T., Shimada A., Taniguchi R. (2017). Analytics of deep neural network in change detection. Proceedings of the 14th IEEE International Conference on Advanced Video and Signal Based Surveillance, AVSS 2017.

[25] Glorot X., Bengio Y. Understanding the difficulty of training deep feedforward neural networks. Proceedings of the Thirteenth International Conference on Artificial Intelligence and Statistics.

[26] Kingma D., Ba J. Adam: A method for stochastic optimization. Proceedings of the 3rd International Conference on Learning Representations (ICLR2015).

[27] Wang Y., Jodoin P.M., Porikli F., Konrad J., Benezeth Y., Ishwar P. CDnet 2014: An expanded change detection benchmark dataset. Proceedings of the 2014 IEEE Conference on Computer Vision and Pattern Recognition Workshops (CVPRW).

[28] Chatfield K., Simonyan K., Vedaldi A., Zisserman A. Return of the Devil in the Details: Delving Deep into Convolutional Nets. Proceedings of the British Machine Vision Conference.

[29] Russakovsky O., Deng J., Su H., Krause J., Satheesh S., Ma S., Huang Z., Karpathy A., Khosla A., Bernstein M. (2015). ImageNet Large Scale Visual Recognition Challenge. Int. J. Comput. Vis..

[30] Fischler M.A., Bolles R.C. (1981). Random sample consensus: A paradigm for model fitting with applications to image analysis and automated cartography. Commun. ACM.

[31] Nishida K., Kurita T. Ransac-svm for large-scale datasets. Proceedings of the 19th International Conference on Pattern Recognition.

